# Growing gold nanostructures for shape-selective cellular uptake

**DOI:** 10.1186/s11671-018-2662-7

**Published:** 2018-08-28

**Authors:** Sulalit Bandyopadhyay, Birgitte H. McDonagh, Gurvinder Singh, Karthik Raghunathan, Axel Sandvig, Ioanna Sandvig, Jens-Petter Andreassen, Wilhelm R. Glomm

**Affiliations:** 1 Ugelstad Laboratory, Department of Chemical Engineering, Norwegian University of Science and Technology (NTNU), N-7491 Trondheim, Norway; 2 Department of Materials Science and Engineering, Norwegian University of Science and Technology (NTNU), N-7491 Trondheim, Norway; 3 Department of Neuroscience, Norwegian University of Science and Technology (NTNU), N-7491 Trondheim, Norway; 4 Division of Pharmacology and Clinical Neurosciences, Department of Neurosurgery, Umeå University, 901 87 Umeå, Sweden; 5 Department of Clinical Neurosciences, University of Cambridge, England, UK; 6 Department of Chemical Engineering, Norwegian University of Science and Technology (NTNU), N-7491 Trondheim, Norway; 7 Polymer Particle and Surface Chemistry Research Group, SINTEF Materials and Chemistry, N-7465 Trondheim, Norway

**Keywords:** Nanomedicine, TEM, Endocytosis, LSPR, Cytotoxicity, Drug delivery, Glioblastoma-astrocytoma

## Abstract

With development in the synthesis of shape- and size-dependent gold (Au) nanostructures (NSs) and their applications in nanomedicine, one of the biggest challenges is to understand the interaction of these shapes with cancer cells. Herein, we study the interaction of Au NSs of five different shapes with glioblastoma-astrocytoma cells. Three different shapes (nanorods, tetrahexahedra, and bipyramids), possessing tunable optical properties, have been synthesized by a single-step seed-mediated growth approach employing binary surfactant mixtures of CTAB and a secondary surfactant. By the use of two-step seed-mediated approach, we obtained new NSs, named nano*makura* (*Makura* is a Japanese word used for pillow) which is reported for the first time here. Spherical Au nanoparticles were prepared by the Turkevich method. To study NS-cell interactions, we functionalized the NSs using thiolated PEG followed by 11-Mercaptoundecanoic acid. The influence of shape and concentration of NSs on the cytotoxicity were assessed with a LIVE/DEAD assay in glioblastoma-astrocytoma cells. Furthermore, the time-dependent uptake of nano*makura* was studied with TEM. Our results indicate that unlike the other shapes studied here, the nano*makura* were taken up both via receptor-mediated endocytosis and macropinocytosis. Thus, from our library of different NSs with similar surface functionality, the shape is found to be an important parameter for cellular uptake.

**Electronic supplementary material:**

The online version of this article (10.1186/s11671-018-2662-7) contains supplementary material, which is available to authorized users.

## Background

Gold nanostructures (NSs) have been used in diverse biomedical applications owing to their shape- and size-dependent optical and electronic properties [1]. Au NSs display modifiable and environmentally sensitive localized surface plasmon resonance (LSPR) [2]. Au LSPR within the visible range makes them suitable candidates for biosensors and good contrast agents for computed tomography (CT) [3, 4] as well as photo-acoustic imaging [5]. NSs with even higher aspect ratios (ARs, defined as the ratio of longitudinal to the transverse dimensions) scatter light more efficiently at the longitudinal plasmon wavelength and can, therefore, perform better in optical imaging applications than spherical NPs. Smaller NSs have enhanced absorption efficiency, yielding improved efficiency in photothermal therapy [6–9]. Also, anisotropic structures have recently been used to form self-assembled structures with superior plasmonic properties that stem from efficient quenching, extremely high molar absorptivities to development of highly localized and intense electromagnetic fields [10–13]. Being able to tune the aspect ratio and size of Au NSs, different structures can be synthesized to cater diverse applications including localized heating, sensing, encapsulating, and releasing target molecules among others [14–16].

Au NSs are typically synthesized using electrotemplating [17], photochemical reduction technique [18], or seeded growth––with or without Ag [19, 20]. In recent years, the seeded growth synthesis has become subject to further modifications, by using organic additives or binary surfactant mixtures to allow control over NS growth [21–25]. The employed synthesis conditions allow for tailoring the properties of the Au NSs by tuning their size and shape, whereby altering the scattering and absorption cross-sections of the NSs. When NSs are introduced in a biological environment, the physiochemical properties of these Au NSs (size, shape, and surface chemistry) play a vital role in cellular uptake, i.e., nanoparticle-cell interaction. Understanding of such interaction is essential to explore new biomedical applications exploiting different shaped Au NSs [26–30]. For example, Au nanorods can be employed to induce cell hyperthermia in cancer cells with the possibility to interfere with cellular functions and in some cases alter them via surface modification of the rods [30–34]. Chen et al. have reported that Au nanocages can be used for targeted photothermal destruction of breast cancer cells [35]. Also, recent reports have demonstrated that the shape of nanoparticles may be equally or more determining for cellular uptake than the size [36, 37]. This necessitates the screening of several shapes (i.e., the interaction of Au NSs of various shapes with cell) under otherwise identical conditions important to describe in vitro*.* To our knowledge, there exists no comprehensive study investigating the interaction of Au NSs of different shapes other than of spheres and nanorods with the cell [38, 39].

Here, we investigate the interactions of five differently shaped Au NSs with glioblastoma-astrocytoma cells and their cellular uptake. Glioblastoma multiforme (GBM) is classified as one of the most aggressive malignant human brain tumors. Patients suffering from GBM have a dismal prognosis, with a mean survival time of less than 15 months with chemotherapy and standard-of-care [40, 41]. Glioblastoma-astrocytoma is especially interesting for uptake studies not only from a medical point of view but also because of their rapid growth. Cell cultures of glioblastoma-astrocytoma have a population doubling time of 32 h and are in continuous need of extracellular nutrients. Due to this, they are highly likely to rapidly engulf foreign objects such as Au NSs, which open up, e.g., for hyperthermic tumor ablation [42], cell labeling, or drug delivery.

In the present work, five different shapes of Au NSs have been synthesized: four anisotropic NSs (nanorods––NRs, nano*makura––*NM, tetrahexahedra––THH, bipyramids––BPs) by a seed-mediated growth approach using binary surfactant mixtures and spherical (SP) particles using a modified Turkevich method [43]. The shape of NSs can be tailored by varying the ratio of two different surfactants. When a two-step seed-mediated growth protocol was followed, Au nano*makura* (*Makura* is Japanese for *pillow*) was synthesized. A two-step surface modification was performed to replace the passivating ligand with 11-mercaptoundecanoic acid (MUA) before the Au NSs were co-incubated with glioblastoma-astrocytoma cells. The effects of shape and concentration on cytotoxicity and uptake of NSs in glioblastoma-astrocytoma cells were assessed.

## Experimental

### Materials

Oleic acid (OA, 90%) was purchased from Alfa Aesar. Silver nitrate (AgNO_3_), didecyldimethylammonium bromide (DDAB, 98%), chloroauric acid (HAuCl_4_.3H_2_O, 99.999%), D-(−)-isoascorbic acid (AsA, 98%), sodium borohydride (NaBH_4,_ ≥ 96%), 11-mercaptoundecanoic acid (MUA, 98%), and O-[2-(3-mercaptopropionylamino) ethyl]-O-ethylpolyethylene glycol (PEG-SH) of molecular weight 5000 Da were purchased from Sigma-Aldrich. Cetyltrimethylammonium bromide (CTAB, 99%+) was purchased from Acros Organics and sodium citrate dihydrate (Na-citrate, ACS grade) from Merck. All chemicals were used as received without further purification. All solutions were prepared using distilled de-ionized water (resistivity ~ 18.2 μΩ-cm) purified by Simplicity® Millipore water purification system.

### Synthesis of anisotropic Au

**Fig. 1:**
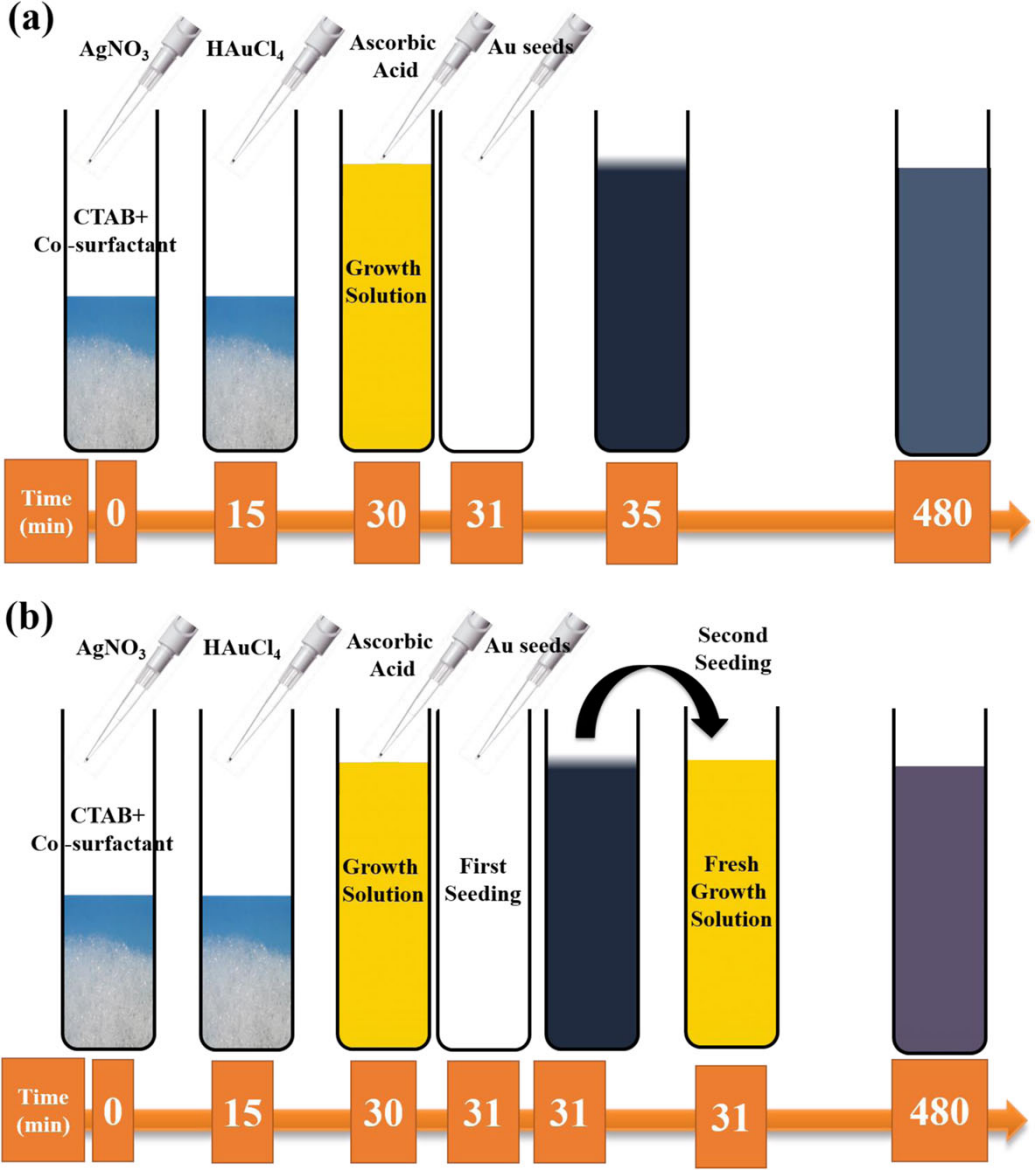
Schematics of synthesis of Au NSs. **a** Schematic showing Ag-assisted seeded growth mechanism used for the synthesis of different shapes of Au NSs. **b** Schematic showing a two-seeded growth mechanism for nano*makura*

Anisotropic Au NSs were synthesized using a Ag-assisted seeded growth method by employing binary surfactants (Table 1). Figure 1a shows a schematic of the synthesis method employed for the growth of NRs, THH, and BPs.

**Table 1:** Moles of CTAB and co-surfactant used for the synthesis of various shapes of Au NS

Sample name	Co-surfactant	Moles of CTAB	Moles of co-surfactant
Nanorods (NRs)	OA	3.3 × 10^−6^	6.3 × 10^− 5^
Tetrahexahedra (THH)	OA	3.3 × 10^−6^	9.4 × 10^−4^
Bipyramids (BPs)	DDAB	3.3 × 10^−6^	4.3 × 10^−4^

In brief, 5 mL of 0.5 mM HAuCl_4_.3H_2_0 was first mixed with 5 mL of 0.2 M CTAB solution and allowed to stir. After that, 1.6 mL of 3.75 mM NaBH_4_ was added to the mixture and allowed to react for 2 min with stirring in order to allow escape of the gas formed during the reaction. The seed solution was used for further growth, after waiting 30 min.

In a typical growth reaction, 15 mL of an aqueous mixture of CTAB and co-surfactant in various ratios was made at 80 °C as reported in Table 1. After cooling the surfactant solution to room temperature, 750 μL of 4 mM solution of AgNO_3_ was added and allowed to stir for 15 min at 35 °C. This was followed by addition of 15 mL of 1 mM HAuCl_4_.3H_2_O solution and allowed to mix under stirring for another 15 min. After that, 135 μL of 0.063 M AsA and 96 μL of Au seeds were added, and the reaction run for 24 h at 35 °C. The products were separated using centrifugation. It is important to note that in the case of OA, the initial yellow color of the growth solution discharges within 15 min (before seed addition) indicating the reduction of Au^3+^ to Au^+^. Figure 1b shows the schematic for the synthesis of the NM, which is based on a two-seeded growth approach. The protocol followed is similar as reported above except for the addition of intermediate growth solution (300 μL) (obtained almost immediately after adding Au seeds to the first growth solution), instead of the regular seeds, to a fresh growth solution and allowing the reaction to continue for 24 h at 35 °C.

### Synthesis of spherical Au NSs

Spherical Au NSs were synthesized using a modified Turkevich method [44]. In a typical synthesis, 10 mL of 10 mM sodium citrate solution was added to a 25-mL reaction flask, maintained at 70 °C. Ten milliliters of 1.5 mM chloroauric acid (HAuCl_4_. 3H_2_O) was added dropwise and allowed to react for 20 min under vigorous stirring at 70 °C. The solution turned purplish red around 8 min after the reaction. After that, the solution was cooled down to room temperature, and spherical Au NSs were separated from the unreacted solution, using centrifugation at 14,500 rpm for 10 min.

### Surface functionalization of Au NSs

To exchange the ligands on the surface of the Au NSs, a two-step procedure adapted and modified from Thierry et al. [45] was followed. The concentrations of the as-synthesized NSs was adjusted to 1 mg mL^−1^ before the start of functionalization steps. The first step depends on the introduction of a PEG-SH layer to partially replace the bound CTAB bilayer since thiol has a greater affinity for Au surface [46]. Further, a PEG-SH layer provides steric stabilization to the NS. In the second step, the residual CTAB is replaced, and the PEG-SH layer is further exchanged with alkanethiol, MUA. MUA allows for a complete removal of CTAB from sterically hindered PEG-SH-coated Au surface.

In a typical functionalization procedure, 1 mL of 1 mg mL^−1^ solution of the Au NS was mixed with 1 mL of 1 mg mL^−1^ solution of the PEG-SH solution. The mixed solution was kept under vigorous stirring allowing the partial replacement of CTAB with PEG-SH for 2 h. After that, PEGylated NSs were removed by centrifugation at 14,500 rpm for 20 min and redispersed in 1 mL of MQ water. To functionalize the Au NSs with carboxylic acid groups, 500 μL of the PEGylated NS solution was mixed with 250 μL of a 10 mM solution of MUA prepared in ethanol/water and allowed to react in a sonic bath maintained at 55 °C for 1 h. After that, the MUA-coated Au NSs were separated from the free MUA using centrifugation at 14,500 rpm for 20 min. MUA-coated Au NSs were easily redispersed in MQ water.

### In vitro studies

#### Glioblastoma-astrocytoma cell culture

Human glioblastoma-astrocytoma cells (U-87 MG, ECACC, Sigma-Aldrich, Salisbury, UK) were cultured in Eagle’s Minimal Essential Medium (EMEM) with 1.25% gentamicin (Sigma) and 10% fetal bovine serum (Autogen Bioclear, Wiltshire, UK). The cultures were supplemented with 2 mM L-glutamine, 1% non-essential amino acids (NEAA, Sigma), and 1 mM sodium pyruvate (NaP, Sigma).

#### LIVE/DEAD® assay

A LIVE/DEAD-cell viability assay (Invitrogen, Life Technologies) evaluates the membrane integrity of cells and consists of two different dyes: calcein AM (excitation/emission 494/517 nm) and ethidium homodimer-1 (excitation/emission 517/617 nm). In live cells, intracellular esterases react with calcein AM and yield a cytoplasmic green fluorescence. Ethidium homodimer-1 (EthD-1) diffuses over damaged cell membranes of dead cells, where it binds to nucleic acids and emits red fluorescence. After labeling with NSs, LIVE/DEAD®-cell viability was performed on glioblastoma-astrocytoma cells as described by the manufacturer. Briefly, a LIVE/DEAD® solution was prepared in 4.5 mL PBS with 2.7 μL calcein (Invitrogen), and 12 μL ethidium homodimer (Invitrogen) was added at 1:1 (*v*/*v*) ratio and left to react for 30 min at 37 °C before microscopy. A nuclear stain (Hoechst 33258, excitation/emission 356/465 nm, Sigma) was added (200 μg mL^−1^) in order to visualize the nucleus and elucidate any nuclear uptake of Au NS. Imaging was performed on an Axiovert 200 M fluorescent microscope (Zeiss, Germany), at × 40 or × 10 magnifications, using AxioVision Rel. 4.3 software. Images were later processed with ImageJ 1.46.

#### Assessing cellular toxicity based on concentration

Cells at 70% confluency were labeled with Au NS at concentrations of NS/media volume of 100 μg mL^−1^, 200 μg mL^−1^, 500 μg mL^−1^, and 2 mg mL^−1^ and incubated at 37 °C for 24 h in 9-well plates (Corning®). Three parallels (wells) were prepared for each concentration. Unlabeled glioblastoma-astrocytoma cultures, at the same stage of confluence, were used as controls. The percentages of dead cells were calculated by manual counting. The highly unordered morphology of glioblastoma-astrocytoma cells makes automated counting less reliable. Three superimposed images of live and dead cells were taken in each well, and the average live and dead cells were calculated for each shape. The same was done for the blank sample, and the viability was assessed by subtracting the average dead/live of the blank.

#### Assessing cellular uptake as a function of time for nano*makura* Au NS

Cells at 70% confluency were labeled with nano*makura* Au NS at concentrations of NS/media volume of 2 mg mL^−1^ and incubated at 37 °C for 2, 6, 12, and 24 h before LIVE/DEAD® assay with nuclear stain. Unlabeled glioblastoma-astrocytoma cultures, at the same stage of confluence, were used as controls. Cell pellets were prepared for TEM via trypsination and centrifugation before primary fixation in paraformaldehyde (2% *v*/*v*) and glutaraldehyde (2.5% *v*/*v*) in PBS (0.1 M, pH = 7.4) overnight. For secondary fixation, two different fixatives were prepared for optimal staining of intracellular membranes. Both were prepared in 0.1 M cacodylate buffer, one containing 1% osmium tetroxide (*v*/*v*) and the other containing 1% osmium tetroxide (*v*/*v*) and 1.5% potassium ferrocyanide (*v*/*v*). One hour after secondary fixation at room temperature, a stepwise dehydration with alcohol was performed, before dehydration with propylene oxide, infiltration, and ultramicrotome sectioning of 70 nm slices.

### Characterization techniques

Bright field (BF) STEM images were acquired using a Hitachi S-5500 electron microscope operating at 30 kV accelerating voltage. High-resolution transmission electron microscopy (HRTEM) images were acquired using JEOL 2100 operating at 200 kV. The size distributions and zeta potentials of the NSs were measured using a Malvern Zetasizer Nano-ZS instrument and the manufacturer’s own software. Dynamic light scattering (DLS) measurements are based on spherical particle assumption and are not appropriate for measuring the hydrodynamic sizes of anisotropic NSs without a multi-angle setup and rigorous fitting of the resulting data. However, DLS has been used in this study as a means to qualitatively track changes in size emanating from the functionalization procedure. MQ water was used as the solvent in all cases. Ultraviolet-visible (UV-Vis) spectra were acquired with a UV-2401PC (Shimadzu) spectrophotometer. The spectra were collected over the spectral range from 200 to 800 nm. X-ray photoelectron spectroscopy (XPS) analyses were performed using a Kratos Axis Ultra DLD spectrometer (Kratos Analytical, UK), equipped with a monochromatized aluminum X-ray source (Al, hν = 1486.6 eV) operating at 10 mA and 15 kV (150 W). Survey spectra were collected over the range of 0–1100 eV binding energy with analyzer pass energy of 160 eV. A hybrid lens (electrostatic and magnetic) mode was employed along with an analysis area of approximately 300 μm × 700 μm.

## Results and discussion

### Synthesis and characterization of anisotropic Au NSs

**Fig. 2:**
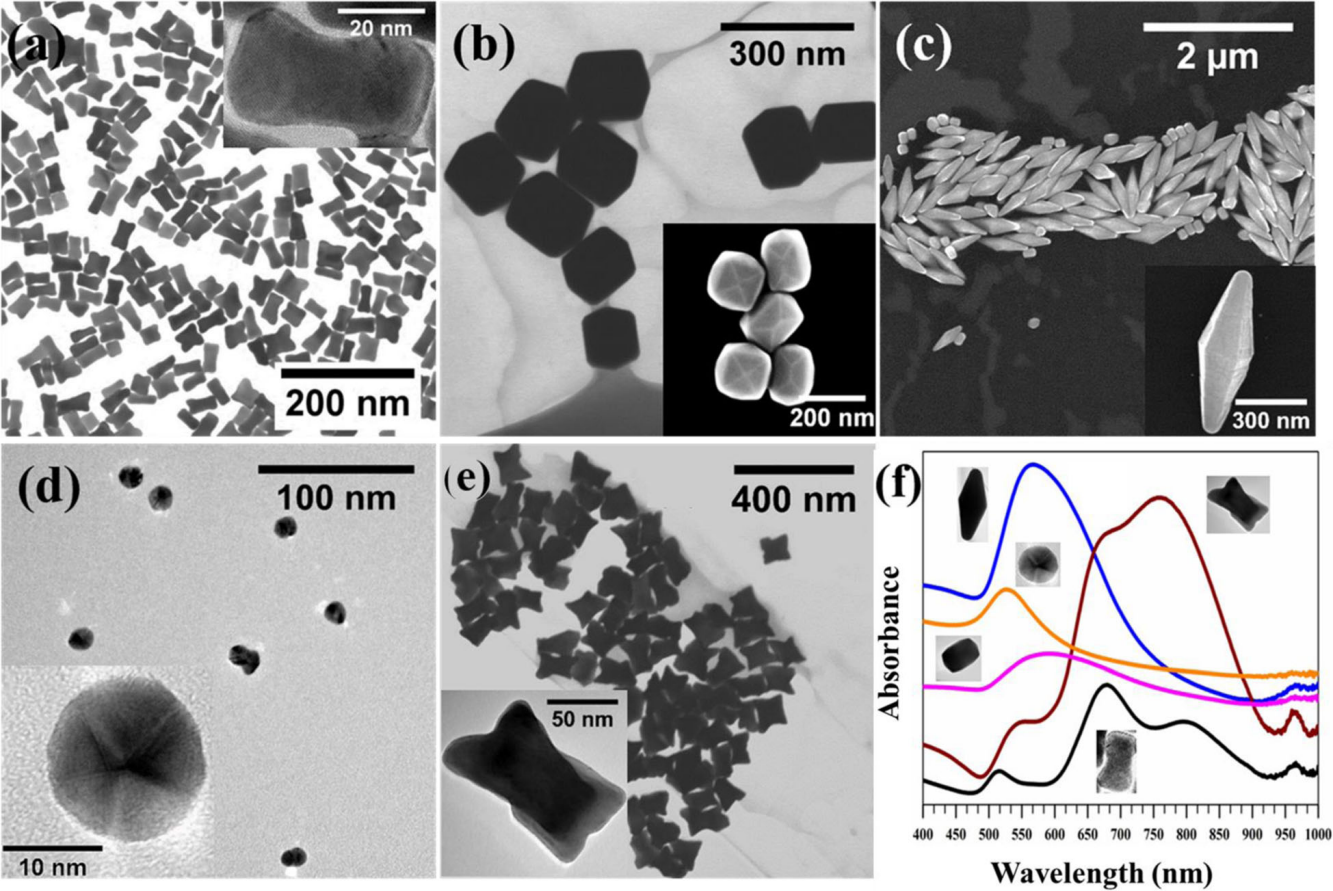
STEM images and UV-Vis spectra of Au NS s of different shapes. **a** BF-STEM image of Au NRs of dog-bone morphology and HRTEM image in inset shows the single crystalline nature of NRs. **b** BF-STEM image of elongated tetrahexahedral Au NSs (inset is SEM image). **c** SEM image of Au BPs (inset is SEM image of single BP). **d** TEM image of Au spherical particles (HRTEM image in inset shows the polycrystalline nature of Au particles). **e** BF-STEM image of Au NMs. **f** UV-vis spectra of Au NSs of different shapes

Anisotropic Au NSs were synthesized via a seed-mediated growth approach employing binary surfactant mixtures. When CTAB capped Au seeds (~ 5 nm) was added to the growth solution of binary surfactant mixtures (molar ratio of OA CTAB ~ 20:1), low aspect ratio Au NRs formed (Fig. 2a, and Table 2). HRTEM image revealed the single crystalline and dog-bone morphology of NRs (inset in Fig. 2a). OA, a fatty acid that also acts as a weak reducing agent facilitates the reduction of Au^3+^ to Au^+^. The change in the color of growth solution from yellow to transparent (~ 15 min) confirmed our observation. Further addition of ascorbic acid to the growth solution increases the reduction rate of Au^+^. As a result, Au atoms diffuse rapidly on end {111} [16] facets of the NRs because the packing of mixed micelle structures are less dense compared to the side {110} and end {100} of the NRs [25]. Therefore, the overgrowth of NRs at the end {111} facets than to side {110} and {100} facets leads to the formation of NRs of dog-bone morphology. Our results also suggest that {110} facets are unlikely to be coated with Au because of the strong interaction of these facets with surfactant molecules compared to other facets. We also confirmed the role of OA and ascorbic acid in the formation of Au NRs of dog-bone morphology. When the concentration of OA or ascorbic acid was decreased in the growth solution, only Au NRs were obtained. These results indicate the decrease in the reduction rate and diffusion rate of gold atoms, leading to the formation of Au NRs (Additional file 1: Figure S1, ESI†).

**Table 2:** Size distribution analysis of Au NSs of different shapes. The average size of NSs was determined from TEM images counting over 100 NSs (Additional file 1: Figure S2, ESI†). The aspect ratio (AR) is calculated as the ratio of the long axis to the short axis

Shape	Long axis (nm)	Short axis (nm)	AR
Nanorods (NRs)	45 ± 8	18 ± 6	2.8 ± 0.7
Tetrahexahedra (THH)	180 ± 25	129 ± 27	1.4 ± 0.3
Nano*makura* (NM)	108 ± 15	71 ± 12	1.6 ± 0.3
Bipyramids (BPs)	644 ± 85	266 ± 19	2.4 ± 0.3
Spheres (SPs)	15 ± 4	15 ± 4	1.0 ± 0.0

When the concentration of OA relative to CTAB was increased in the growth solution (Table 1), Au NSs of elongated THH shape were obtained (Fig. 2b and Table 2). The change in the shape of Au NSs can be explained based on the modification of mixed micelle structures by OA. The rod-like mixed micelle structures are formed at a low concentration of OA. The increased amount of OA in the mixed micelle structures modifies its structure to convex and facilitate the formation of elongated THH Au NSs. Our previous study also revealed that an increase in the concentration of co-surfactant makes the mixed micelle structures more convex [25]. The shape of Au NSs can also be changed by replacing OA with DDAB. We obtained Au NSs of bipyramid (BP) shape by the use of CTAB and DDAB (Fig. 2c and Table 2) as reported in our previous work [25]. Further, spherical Au particles were synthesized by a modified Turkevich method (Fig. 2d).

**Fig. 3:**
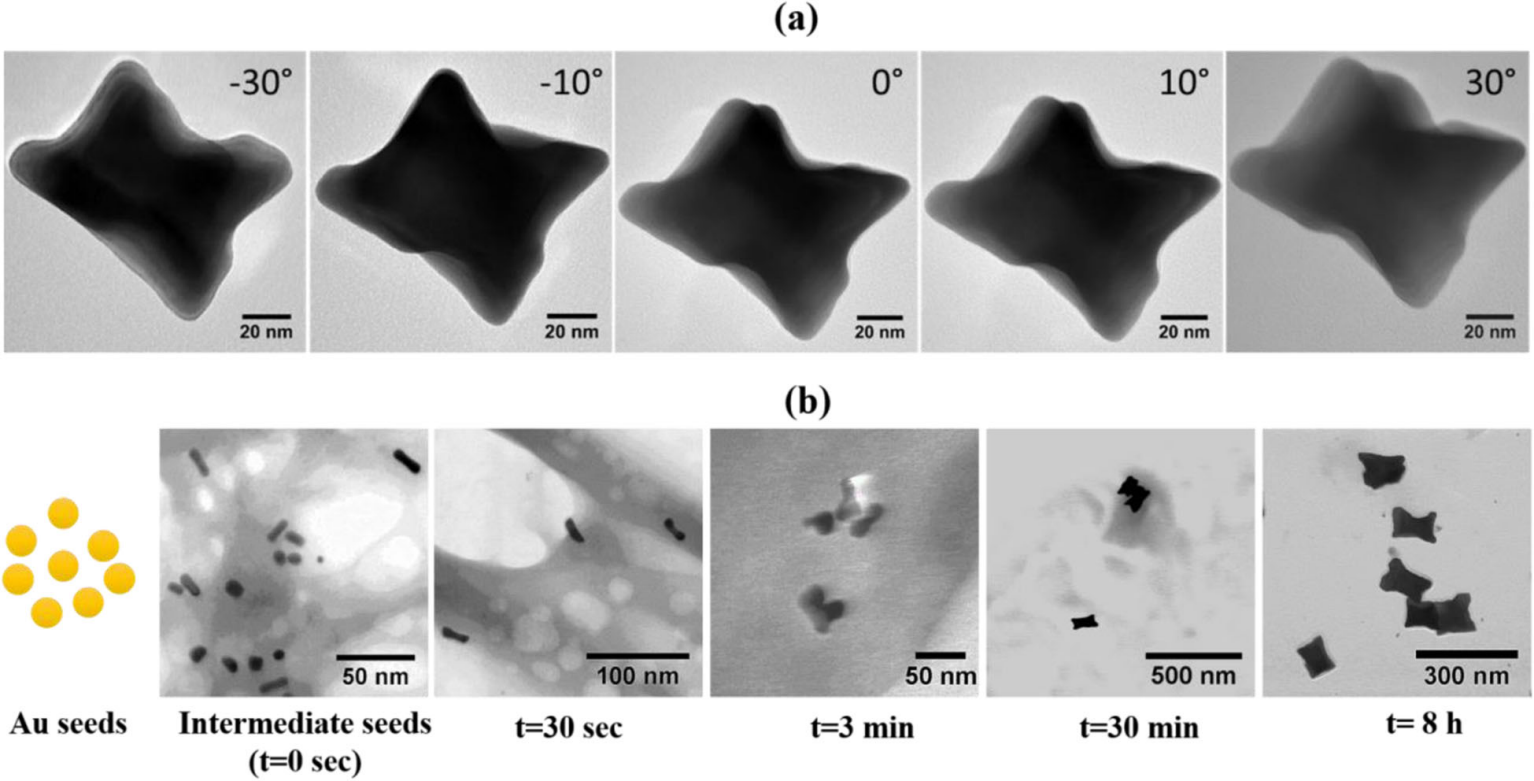
Morphology and growth of Au NM (nano*makura*). **a** TEM images of NM taken at different angles. **b** BF-STEM images show the growth steps in the formation of NM type Au NSs. The solution taken from the growth solution at different time points was added directly to TEM grid without purification

We also investigated the influence of seed solution on the shape of Au NSs. The seed solution was taken from the growth solution of CTAB and OA (OA:CTAB ~ 20:1) after 1 min of the reaction and added to the fresh growth solution containing OA:CTAB ~ 20:1. Au NSs of nano*makura* (NM) morphology were obtained after the completion of growth reaction (Fig. 2e). The morphology of NM appears similar to dog-bone. However, since the NSs grow in all directions as shown in TEM images taken at different angles (Fig. 3a and Table 2), therefore, we call these NSs as NM. To illustrate the growth mechanism of Au NMs, a small volume was taken from the growth solution at different time intervals and the intermediate reaction was analyzed using STEM imaging (Fig. 3b). When CTAB-coated seed particles were added to the first growth solution, the color of growth solution turned rapidly into dark violet indicating the formation of anisotropic Au NSs. A 300 μL of solution from the first growth solution was added to the second growth solution, and afterward, few drops of the solution were added to TEM grid immediately.

Representative STEM images showed relatively faster growth of Au NM in the longitudinal direction than to transverse (0 s). After 30 s, NSs resembling bow-tie configuration were seen. NM having final sizes were already observed around 3 min of the reaction. We did not see any further change in the shape and size of NSs after 30 min and 8 h. Based on our analysis, the overall growth of Au NMs can be hypothesized to be following a stochastic, “popcorn”-like autocatalytic growth mechanism, in which individual seeds lie dormant for some time before suddenly and rapidly growing into the final shapes, as has been observed for NRs by Cortie et al. [47]. The NMs have a three-dimensional structure, shown by HRTEM images of the NMs obtained at different rotation angles (Fig. 3a) unlike previously reported dog-bone-shaped NSs [48, 49].

The optical properties of the Au NSs of different shapes were measured, and the results are shown in Fig. 2f. Au NSs display tunable LSPR characteristics over the UV-Vis––visible––near-infrared (IR) range. The plasmon bands split up into multiplets for anisotropic structures––the longitudinal and transverse bands, owing to resonance oscillations along different axes. NRs show at least three distinct bands––516 nm, 679 nm, and 796 nm, the strongest being the middle one. The emergence of a third band can be associated with the polydispersity of the NRs caused due to the etching effect of oleic acid. This leads to the formation of nanorods with rough edges. Both transverse and longitudinal resonance peaks (557 and 760 nm, respectively) are observed for NM, which has a more jagged surface than the nanorods. However, for larger structures (THH and BPs), single and broad LSPR peaks are observed at 568 nm and 593 nm, respectively. While the UV-vis spectra for THH show similar resemblance to previous studies [50], the two modes for BPs seem to be fused into a broad peak unlike otherwise observed [51]. This can be attributed to a low yield of the BP shape or non-shape-selective centrifugation applied to the Au NS or an uneven coating leading to shape anisotropy in solution.

### Surface functionalization of Au NSs

**Fig. 4:**
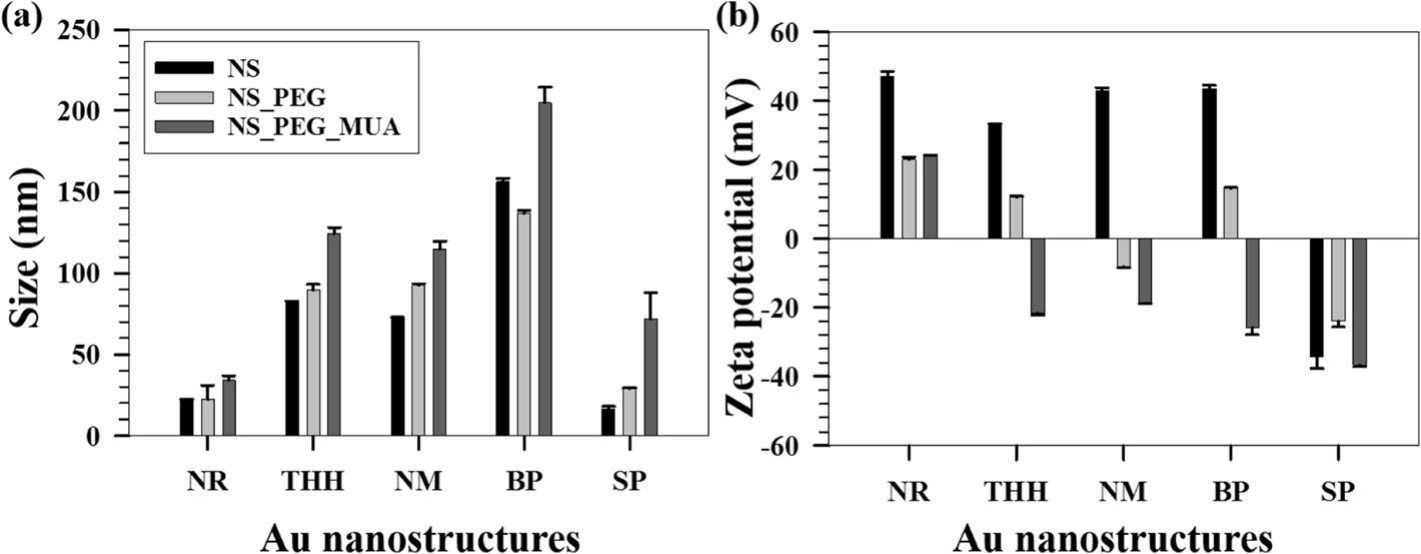
Sizes and zeta potentials of Au NSs. **a** Variation of DLS sizes of the Au NSs after each stage of functionalization. **b** Variation of zeta potentials of Au NSs with each stage of functionalization. *X*-axis represents Au nanostructure of different shapes (NR nanorods, THH tetrahexahedra, NM nano*makura*, BP bipyramid, SP spherical)

Au NSs of different shapes were functionalized using a two-step method––replacing the bilayer CTAB with O-[2-(3-mercaptopropionylamino) ethyl]-O-methylpolyethylene glycol (PEG-SH) and subsequently with MUA. Figure 4a shows the hydrodynamic diameters of the NSs at each stage of the functionalization. A sequential increase in the sizes is obtained when compared to CTAB-coated NSs, for each NS (except for BPs) indicating successful functionalization. As DLS measurements are based on spherical particle assumption, the size determination analysis for the anisotropic NSs must be approximated to spherical NSs having the same diffusion coefficients as that of the anisotropic NSs.

This clarifies a slight decrease in size for PEG-SH-coated BPs and also supports the UV-vis data above. As a result of the functionalization, the surface charges of the NSs decrease massively as displayed in Fig. 4b. The cationic surfactant gets readily displaced with PEG-SH, which is further replaced by MUA owing to higher affinity towards Au surface. Owing to the small size of MUA, it has higher flexibility to displace CTAB that remains on the NSs, even after PEGylation. Final zeta potential values of MUA-coated NSs reflect negatively charged surfaces for all NSs except for NRs. This discrepancy can be linked to the uneven coating of the small NRs or their polydispersity, and the measurement principle applied. For the spherical NSs, the initial negative surface charge is due to citrate coating. However, high magnitudes of the zeta potentials for all the NSs ensure the stability of the NSs in aqueous solutions. XPS measurements carried out on the Au NSs after each stage of functionalization, i.e., with PEG-SH followed by MUA shows a very low content of bromine on the surface, confirming removal of bound CTAB in large amounts from the surfaces of the NSs. (Table 1, ESI).

Further, a coating of the NSs with PEG-SH and MUA does not change their optical characteristics dramatically. However, sequential peak broadening is obtained after functionalization for the anisotropic NSs (Additional file 1: Figure S3, ESI†). This can be because of different axes of rotations of the NSs due to anisotropy, non-uniform coating, size enlargement (DLS data), a polydispersity of the samples, or a combination of the above. As the optical properties of Au NSs depend on shape, surface, size, and aggregation state, so do their interactions with cells. Cell interaction studies can reveal to which extent Au NSs are taken up, their cytotoxic effects, and can point to future therapeutic and diagnostic applications.

### Cellular interaction of Au NSs of different shapes

Generally, Au NSs enter the cell through endocytosis, which can be receptor-mediated or receptor-independent, through actin-dependent phagocytosis, or through other currently unknown endocytic routes [52]. Describing which route the NS takes is of critical importance, as it ultimately determines the NS intracellular fate [53]. Studies have shown that the mechanism of intracellular uptake depends on the physicochemical properties, AR, and the surface characteristics of the NS, as well as the cell type [54–58]. The charge of the surface stabilizing molecules will effectively be the charge of the NS [59]. Positively charged NSs have a strong protein adsorption in biological media and can severely damage the membranes of cells. Due to this, a neutral or negative charge is preferable to avoid strong adsorption on cell membranes and/or protein adsorption [26, 60]. Furthermore, the shape of NSs will determine to which extent the surface coverage of stabilizing molecules is uniform or not [61].

Here, all Au NSs, except the spherical NSs, were synthesized with the surface active agent CTAB. Au NSs were functionalized with MUA to gain a negatively charged surface prior to the cell interaction studies. Stable MUA-coated Au NSs were subsequently co-incubated with human glioblastoma-astrocytoma cells for 24 h, and the effect of shape and concentration on cytotoxicity was assessed with a LIVE/DEAD® assay, supplemented with a nuclear stain to highlight intracellular Au NS.

**Fig. 5:**
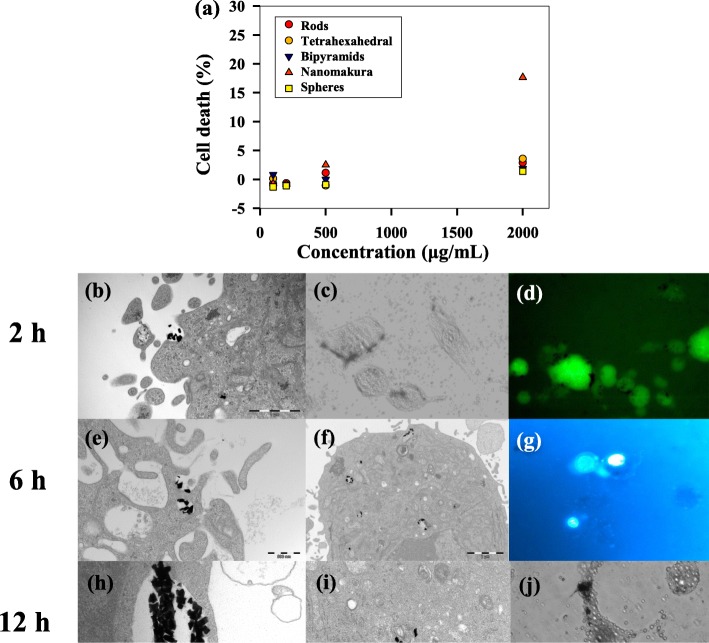
Effect of Au NSs on glioblastoma-astrocytoma cells. **a** Percentage cell death of glioblastoma-astrocytoma cells as a function of concentration of AuNSs. Incubation time was 24 h. Images **b**–m are acquired after incubation of *makura*-shaped AuNSs in glioblastoma-astrocytoma cells taken at several time points up to 24 h. **b** TEM shows an invagination of the cellular membrane (m membrane, scale bar = 1 μm), and **c** halogen and **d** fluorescence images show association of makura-shaped AuNSs at the cellular membrane. **e** Uptake of makura-shaped AuNSs were observed after 6 h (scale bar = 500 nm), **f** with uptake in intracellular vesicles (v vesicle, scale bar = 2 μm). **g** Staining of the nucleus (*n*) suggests that makura-shaped Au NSs were excluded from the nucleus. **h** TEM images taken after 12 h suggest uptake via macropinocytosis (scale bar = 500 nm), with **i** intravesicular location of makura-shaped AuNSs (vm vesicular membrane, scale bar = 500 nm). **j** Intracellular compartmentalization was also visible from the microscopy. Uptake of makura-shaped AuNSs continued at 24 h as seen in TEM images **k** (scale bar = 2 μm) and **l** (scale bar = 5 μm). **m** Detachment of glioblastoma-astrocytoma cells from the surface was observed, which most likely is an indication of cell death

The highest cell death was observed with the NM at high concentration (Fig. 5a), with a cell death close to 20% after blank correction. The other NSs did not show the same trend in cytotoxicity, which might indicate that NMs are taken up at a higher rate/volume than the other shapes [62]. Cell counting for these shapes showed a cytotoxicity below 5% after blank correction (for images from all shapes, see Additional file 1: Figure S4, ESI†).

The data presented here suggest that size plays a minor role in cytotoxicity in glioblastoma-astrocytoma cells. For instance, the small spherical NSs (15 nm) showed the same uptake/cytotoxicity as the large BPs (650/270 nm). Based on previous studies, we expected the NRs to give the highest cytotoxicity, due to their AR and surface charge. Positively charged NSs are considered to be particularly toxic as they can induce apoptosis [63] and cause the production of reactive oxygen species [64]. However, in the data presented here, the positively charged NRs did not show increased cell death compared to any other shape (Fig. 5a). This might be explained as follows: the surface charge of NS was determined with zeta potential measurements, an approach based on spherical particle assumption (Table 2). Thus, the reported charge of the NRs most likely misrepresents their actual charge. Although the AR of the rods synthesized here is large (2.8), their overall size falls in a size range that shows good uptake in cells (20–50 nm). [30, 65] A previous study has suggested that the size of NS does not only seem to govern endocytosis but also exocytosis. For instance, the removal half-life of 14 nm AuNS was much faster than removal half-life of 74 nm AuNS [26]. Here, the smaller Au rods and spheres, may, therefore, have been removed via exocytosis, which may explain their low cytotoxicity.

The main feature of the NM is not their size, but their irregular structure, and it appears from the image in Fig. 5a that shape has been the determining factor for the high uptake. However, an irregular morphology may have undermined the surface coverage of MUA and as such decreased the solution stability of the NM. Also, we cannot exclude that as two-dimensional cell studies are affected by gravity, a low stability in the cell medium increases the likelihood of sedimentation which can propel the endocytosis.

To get further insight into the uptake mechanism and interactions, we followed uptake of NM at high concentration (2 mg mL^−1^) with light microscopy and TEM for 24 h. The images show that after 2 h of co-incubation, the NM associate with the cellular membrane (Fig. 5c, d) and are engulfed by the cells (Fig. 5b, e). The mechanism of endocytosis seems to be initially receptor-mediated (Fig. 5b) and at later stages through macropinocytosis (Fig. 5h). The latter is believed to occur when large objects enter a cell, and the aggregation of NSs may have induced macropinocytosis. This might mean that the initial solution stability of the Au NM is sufficient, but that with time they aggregate and are taken up as larger species, most likely due to protein adsorption. Uptake of any extracellular NS in an intracellular vesicle involves wrapping of the cell membrane. If many such wrapping events occur, this alters the global elasticity of the cell membrane, which in turn affects the membrane integrity. If many macropinocytosis events occur, the membrane integrity may be severely impaired. This is believed to be one of the effects for the cytotoxicity observed for the NM.

Once inside the cell, it appears that the Au NM align at the periphery of the vesicles, adhering to the vesicular membrane (Fig. 5i). The endosome seems to be trafficked towards the nucleus (Fig. 5f, g, and l), which is consistent with previous studies that show that Au NSs taken up via receptor-mediated endocytosis may eventually end up in the Golgi apparatus [53, 66]. The uptake appears to continue upto 24 h (Fig. 5k), owing to the high concentration gradient of Au NS in the cell culture media [59].

At 24 h with co-incubation, the morphology of the cells changes (Fig. 5m), going from star-shaped to a more rounded shape with less visible filopodia [67], followed by detachment from the surface. Although this study does not go further in the molecular events following uptake of NS, a detachment of filopodia may suggest that NM can interact with the cytoskeleton and cause detachment and apoptosis.

We extended the cytotoxicity assay beyond 24 h and investigated the result of co-incubation at 48 and 72 h, the reason being that few cell studies are performed at these time points [68, 69], and the main argument being that cellular uptake reaches a plateau at 24 h [70]. Our results show (Additional file 1: Figure S5, ESI†) that beyond 24 h, the cells continued to detach from the flask surface, i.e., cell death and uptake continue beyond 24 h. As we cannot exclude that starvation of the cells would have increased the cytotoxicity and detachment, a cytotoxic response is a dynamic process likely to evolve over time differentially.

## Conclusions

In summary, we have synthesized five different shapes of Au NSs using a seed-mediated growth approach (nanorods, nano*makura*, tetrahexahedral, bipyramidal) and the Turkevich method (spherical). These NSs have different sizes: the smallest being the NRs and the largest being the BPs, ranging from 22 to 156 nm. Their optical properties measured using UV-vis spectroscopy show LSPR span from UV, visible, to near IR. High values of zeta potential render good stability in aqueous solution. With an aim to exchange the cationic surfactant on the surface, a two-step functionalization protocol was employed to replace the CTAB with PEG thiol and MUA.

After coating, the NSs showed a decrease in surface charge, coupled with an increase in size, proving successful functionalization. In vitro studies were performed for all the synthesized NSs involving co-incubation with glioblastoma-astrocytoma cells for 24 h. The greatest cytotoxic response (~ 20%) was observed with NM at high concentration, which is consistent with higher uptake. An in––depth study with TEM revealed a time-dependent internalization in cancer cells via endocytosis and macropinocytosis. This successful internalization of the Au NM in cancer cells, coupled with their unique physicochemical properties, render them suitable for hyperthermia and drug delivery to cancer cells while being simultaneously imaged.

## Additional file

Additional file 1: **Figure S1.** STEM image of Au NRs grown from the solution of (a) low OA concentration (i.e., OA CTAB ~ 10:1) and (b) low ascorbic acid concentration (60 μL of 63 mM). **Figure S2.** Histograms of differently shaped AuNSs obtained from representative STEM images, showing relative population percentages of (a), (b) nanorods (NRs) (c), (d) tetrahexahedra (THH) (e), (f) nano*makura* (NM) (g), (h) bipyramids (BPs), and (i) spheres (SPs). **Figure S3.** UV-Vis spectra of (a) nanorods (NRs), (b) tetrahexahedra (THH), (c) nano*makura* (NM), (d) bipyramids (BPs), and (e) spheres (SPs) after each stage of functionalization. **Figure S4.** Superimposed images of differently shaped AuNSs after 24-hour co-incubation with glioblastoma-astrocytoma cells. The column to the far left show the halogen images, the middle column shows the cells stained with calcein, while the column to the far right show the superimposed images taken of the cell nuclei (white). Images (a)–(c) show glioblastoma cells incubated with nanorods (NRs), (d)–(f) spheres (SPs), (g)–(i) bipyramids (BPs), (j)–(l) tetrahexahedra (THH), and (m)–(o) nano*makura* (NM). **Figure S5.** Images of nano*makura* NSs co-incubated with glioblastoma-astrocytoma cells as a function of time. The column to the far left shows the halogen images; the middle column shows the cells stained with calcein, except image q. The column to the far right shows the superimposed images taken of the cell nuclei (white). Images (a)–(c) were taken after 2 h, (d)–(f) 6 h, (g)–(i) 12 h, (j)–(l) 24 h, (m)–(o): 48 h, and (p)–(r): 72 h of co-incubation. Images (s)–(u) show glioblastoma-astrocytoma cells not incubated with any NSs. (DOCX 2861 kb)

## Abbreviations

AgNO_3_: Silver nitrate
Au: Gold
BF: Bright filed
BPs: Bipyramids
CTAB: Cetyltrimethylammonium bromide
DDAB: Didecyldimethylammonium bromide
DLS: Dynamic light scattering
EMEM: Eagle’s Minimal Essential Medium
EthD-1: Ethidium homodimer-1
GBM: Glioblastoma multiforme
HAuCl4: Cholorauric aicd
HRTEM: High-resolution transmission electron microscopy
IR: Infrared
LSPR: Localized surface plasmon resonance
MUA: 11-Mercaptoundecanoic acid
Na-citrate: Sodium citrate dihydrate
NM: Nano*makura*
NRs: Nanorods
Ns: Nanostructure
OA: Oleic acid
PEG-SH: O-[2-(3-Mercaptopropionylamino) ethyl]-O-ethylpolyethylene glycol
STEM: Scanning transmission electron microscopy
TEM: Transmission electron microscopy
THH: Tetrahexahedra
UV-Vis: Ultraviolet-visible
XPS: X-ray photoelectron spectroscopy
