# Supplementary material for: Growing gold nanostructures for shape-selective cellular uptake
Source: Nanoscale Res Lett. 2018 Aug 28;13:254. doi: 10.1186/s11671-018-2662-7 (PMC6113194; doi:10.1186/s11671-018-2662-7)
Supplement: Supplementary file 1 — Figure S1. STEM image of Au NRs grown from the solution of (a) low OA concentration (i.e., OA CTAB ~ 10:1) and (b) low ascorbic acid concentration (60 μL of 63 mM). Figure S2. Histograms of differently shaped AuNSs obtained from representative STEM images, showing relative population percentages of (a), (b) nanorods (NRs) (c), (d) tetrahexahedra (THH) (e), (f) nanomakura (NM) (g), (h) bipyramids (BPs), and (i) spheres (SPs). Figure S3. UV-Vis spectra of (a) nanorods (NRs), (b) tetrahexahedra (THH), (c) nanomakura (NM), (d) bipyramids (BPs), and (e) spheres (SPs) after each stage of functionalization. Figure S4. Superimposed images of differently shaped AuNSs after 24-hour co-incubation with glioblastoma-astrocytoma cells. The column to the far left show the halogen images, the middle column shows the cells stained with calcein, while the column to the far right show the superimposed images taken of the cell nuclei (white). Images (a)–(c) show glioblastoma cells incubated with nanorods (NRs), (d)–(f) spheres (SPs), (g)–(i) bipyramids (BPs), (j)–(l) tetrahexahedra (THH), and (m)–(o) nanomakura (NM). Figure S5. Images of nanomakura NSs co-incubated with glioblastoma-astrocytoma cells as a function of time. The column to the far left shows the halogen images; the middle column shows the cells stained with calcein, except image q. The column to the far right shows the superimposed images taken of the cell nuclei (white). Images (a)–(c) were taken after 2 h, (d)–(f) 6 h, (g)–(i) 12 h, (j)–(l) 24 h, (m)–(o): 48 h, and (p)–(r): 72 h of co-incubation. Images (s)–(u) show glioblastoma-astrocytoma cells not incubated with any NSs. (DOCX 2861 kb) [file 11671_2018_2662_MOESM1_ESM.docx]

**Supplementary Information**

**Growing gold nanostructures for shape-selective cellular uptake**

Sulalit Bandyopadhyay^a*^, Birgitte H. McDonagh^a^, Gurvinder Singh^b^, Karthik Raghunathan^a^, Axel Sandvig^c,d^, Ioanna Sandvig^c,e^, Jens-Petter Andreassen^f^, Wilhelm R. Glomm^a,g^

*^a^Ugelstad Laboratory, Department of Chemical Engineering, Norwegian University of Science and Technology (NTNU), N-7491, Trondheim, Norway.*

*^b^Department of Materials Science and Engineering, Norwegian University of Science and Technology (NTNU), N-7491, Trondheim, Norway.*

*^c^Department of Neuroscience, Norwegian University of Science and Technology (NTNU), N-7491, Trondheim, Norway.*

*^d^Division of Pharmacology and Clinical Neurosciences, Department of Neurosurgery, Umeå University, 901 87 Umeå, Sweden.*

*^e^Department of Clinical Neurosciences, University of Cambridge, England, UK.*

*^f^Department of Chemical Engineering, Norwegian University of Science and Technology (NTNU), N-7491, Trondheim, Norway.*

*^g^Polymer Particle and Surface Chemistry Research Group, SINTEF Materials and Chemistry, N-7465 Trondheim, Norway.*

Email: sulalit.bandyopadhay@ntnu.no, ratnavo@gmail.com


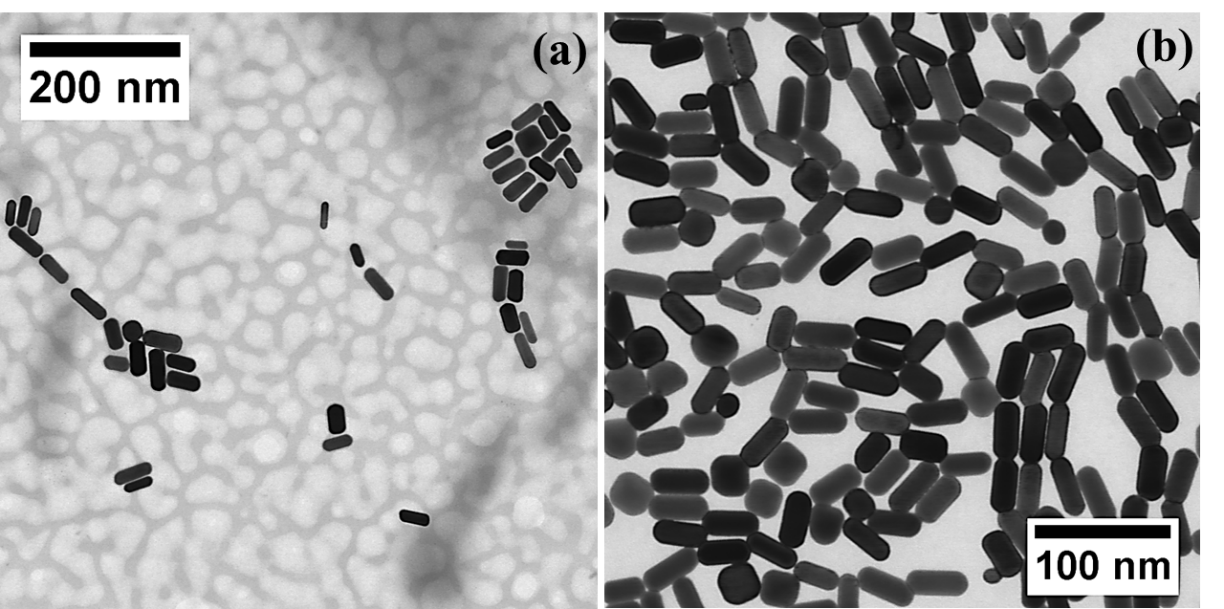


**Fig. S1** STEM image of Au NRs grown from the solution of (a) low OA concentration (i.e., OA: CTAB~ 10:1) and (b) low ascorbic acid concentration (60 µL of 63mM).


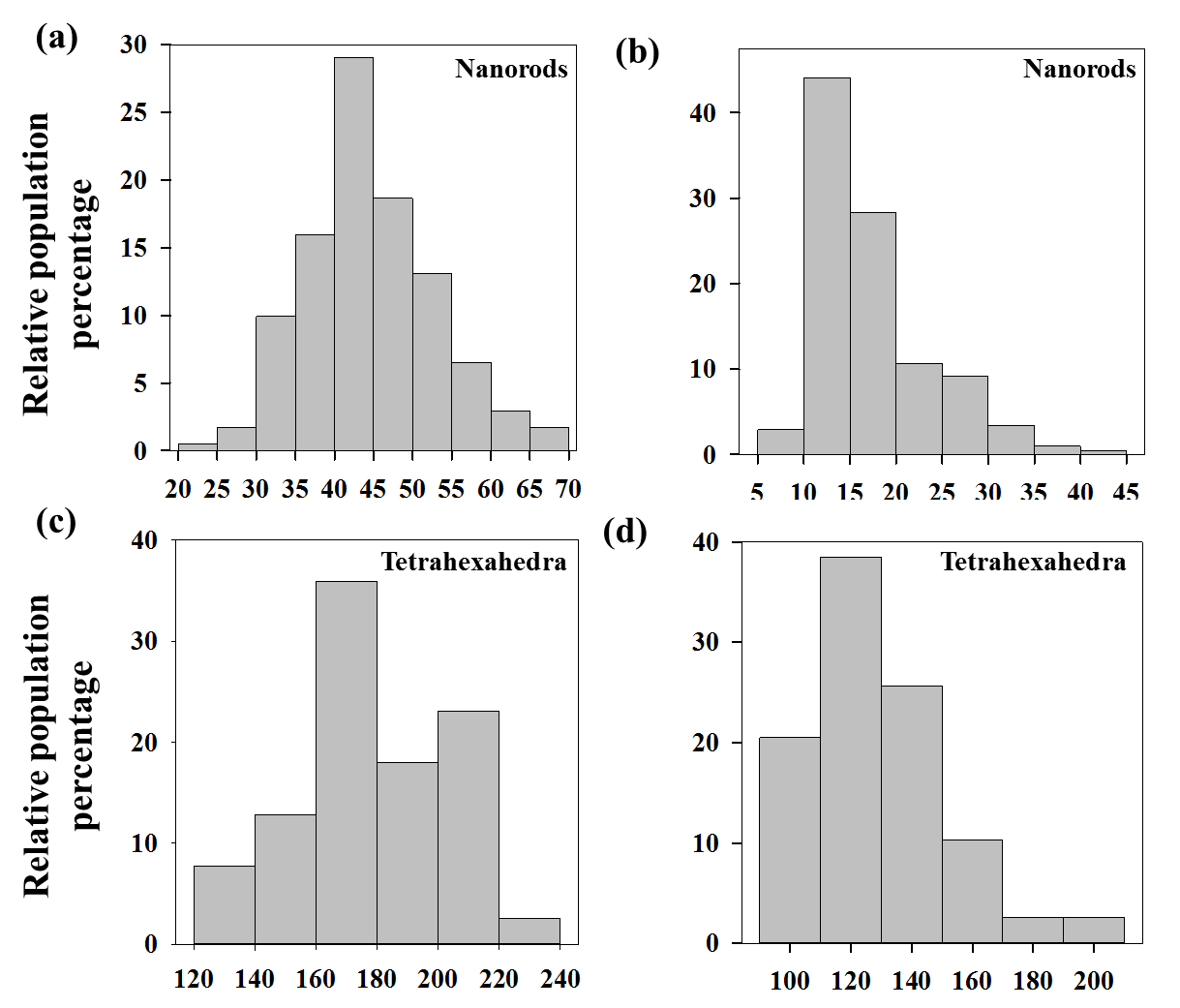

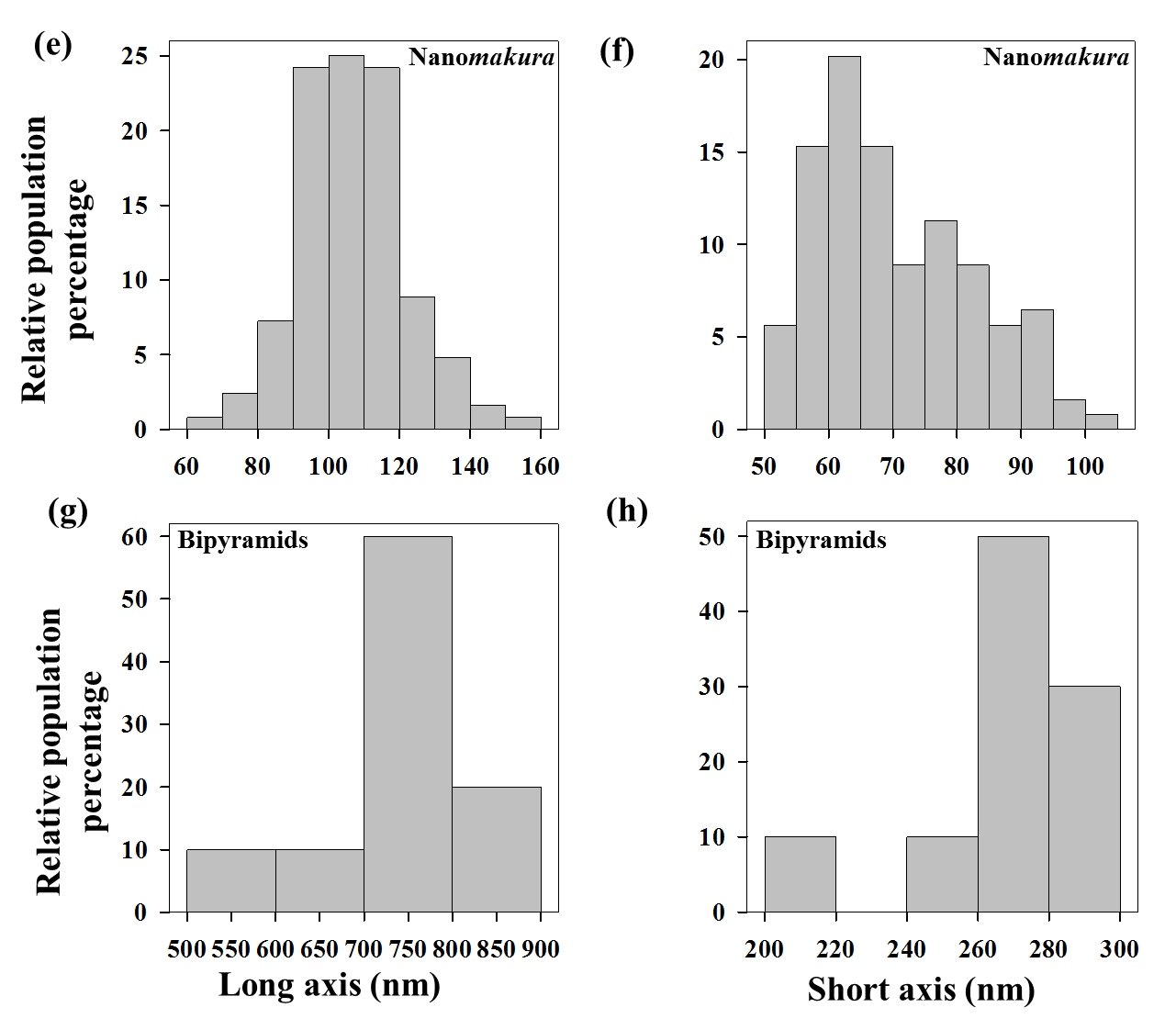

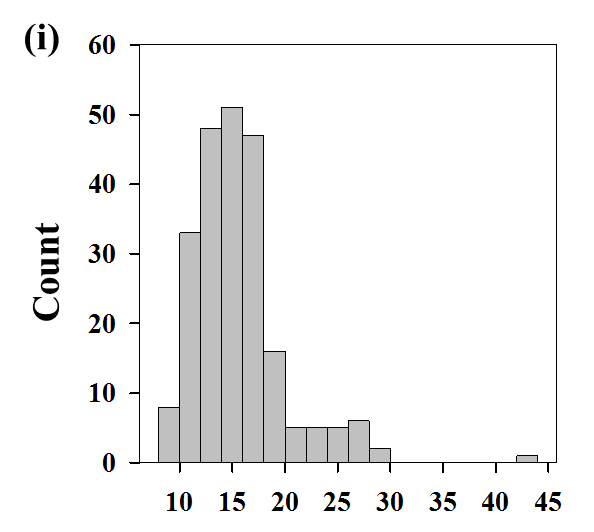


**Fig. S2** Histograms of differently shaped AuNSs obtained from representative STEM images, showing relative population percentages of (a),(b) nanorods (NRs) (c),(d) tetrahexahedra (THH) (e),(f) nanomakura (NM) (g),(h) bipyramids (BPs) and (i) spheres (SPs).


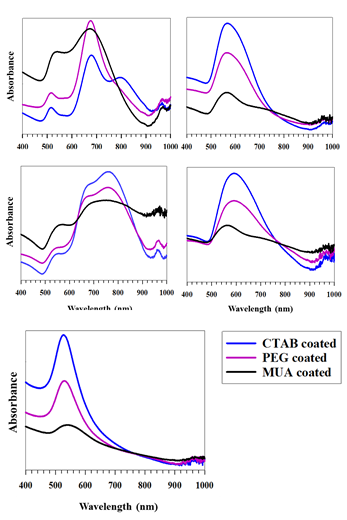


**Fig. S3** UV-Vis spectra of (a) nanorods (NRs) (b) tetrahexahedra (THH) (c) nano*makura* (NM) (d) bipyramids (BPs) and (e) spheres (SPs) after each stage of functionalization.


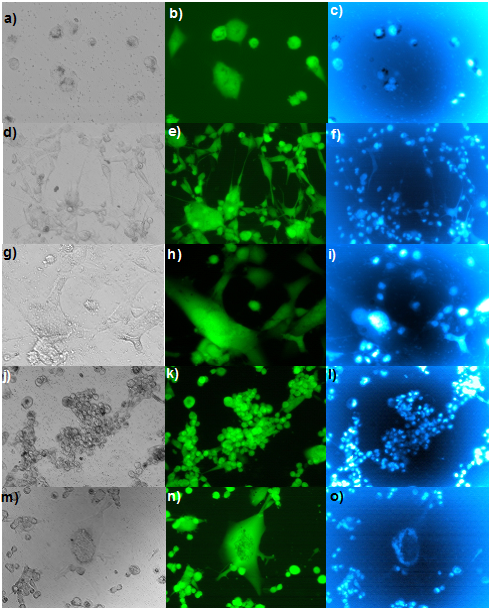


**Fig. S4** Superimposed images of differently shaped AuNSs after 24 hour co-incubation with glioblastoma-astrocytoma cells. The column to the far left show the halogen images, the middle column show the cells stained with calcein, while the column to the far right show the superimposed images taken of the cell nuclei (white). Images (a)-(c) show glioblastoma cells incubated with nanorods (NRs), (d)-(f) spheres (SPs), (g)-(i) bipyramids (BPs), (j)-(l) tetrahexahedra (THH), and (m)-(o) nanomakura (NM).


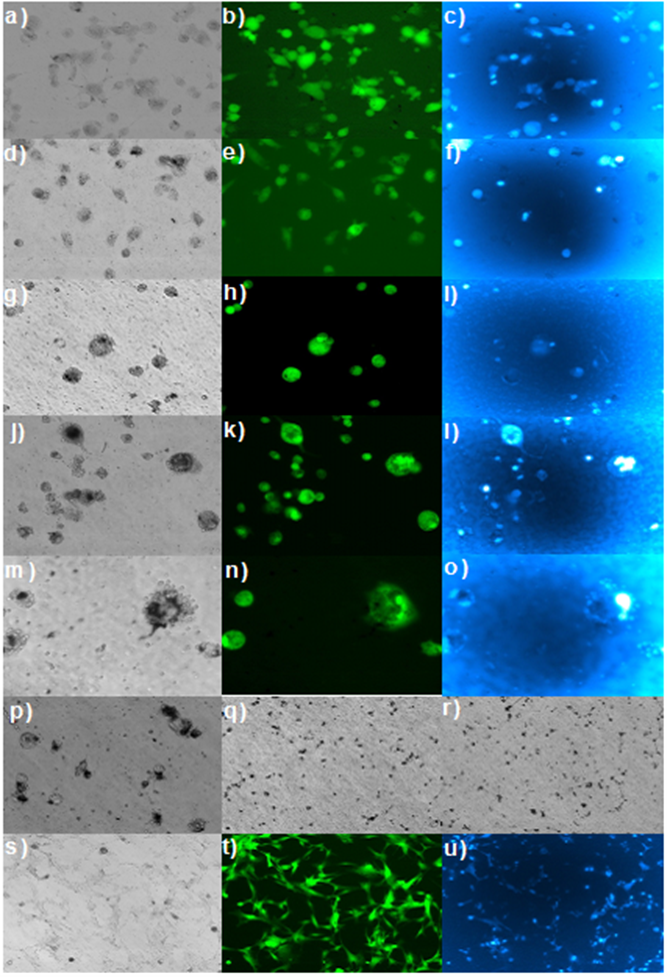


**Fig. S5** Images of nanomakura NSs co-incubated with glioblastoma-astrocytoma cells as a function of time. The column to the far left shows the halogen images, the middle column shows the cells stained with calcein, except image q. The column to the far right shows the superimposed images taken of the cell nuclei (white). Images (a)-(c) were taken after 2 h, (d)-(f) 6 h, (g)-(i) 12 h, (j)-(l) 24 h, (m)-(o): 48 h, and (p)-(r): 72 h of co-incubation. Images (s)-(u) show glioblastoma-astrocytoma cells not incubated with any NSs.

| **Sample** | **C** | **O** | **Br** | **N** | **S** |
| --- | --- | --- | --- | --- | --- |
| Nanomakura​  (NM_CTAB) | 87.9 | 4.1 | 3.5 | 3.9 | 0 |
| Nanomakura​ _PEGSH (NM_PEGSH) | 49.4 | 46.6 | 0.6 | 0 | 1.3 |
| Nanomakura​ _PEGSH_MUA  (NM_PEGSH_MUA) | 70.7 | 20.9 | 0.4 | 0 | 7.9 |
| Spheres  (SP_Citrate) | 57.6 | 41.3 | 0 | 0.8 | 0.6 |
| Spheres_PEGSH  (SP_PEGSH) | 64.4 | 28.6 | 0 | 1.5 | 3.6 |
| Spheres_PEGSH_MUA  (SP_PEGSH_MUA) | 51.8 | 27.9 | 0 | 0 | 17.8 |
| Bipyramids  (BP_CTAB) | 91.3 | 2.3 | 2.9 | 3.4 | 0 |
| Bipyramids_PEGSH  (BP_PEGSH) | 63.4 | 25.6 | 1.8 | 1.4 | 7.7 |
| Bipyramids_PEGSH_MUA  (BP_PEGSH) | 74.7 | 16.5 | 0.7 | 0.5 | 7.9 |
| Tetrahexahedra_PEGSH_MUA  (THH_PEGSH_MUA) | 68.8 | 22.6 | 0.8 | 0 | 7.6 |
| Nanorods_PEGSH_MUA  (NR_PEGSH_MUA) | 58.6 | 29.1 | 1.1 | 0 | 9.5 |
| Nanorods_PEGSH  (NR_PEGSH) | 80.5 | 16.9 | 1.7 | 1.1 | 1.3 |

**Table S1.** Percentage elemental composition of Au NSs determined from XPS survey spectra, before and after functionalization.
